# Supplementary material for: Are carbon emissions trading and green financial instruments synergistic? -Comprehensive quantitative research based on content analysis
Source: PLoS One. 2024 Mar 7;19(3):e0298601. doi: 10.1371/journal.pone.0298601 (PMC10919601; doi:10.1371/journal.pone.0298601)
Supplement: S1 Data — (ZIP) [file pone.0298601.s001.zip › Data/地级市Z_F工作报告.zip!/╜¡╬≈/╕╙╓▌╩╨/└┤╫╘╣1⁄2╓┌║┼í╛┬φ┐╦╩2╛▌═°í┐_2007.docx]

各位代表：

　　现在，我代表市人民政府向大会作工作报告，请予审议，并请市政协各位委员和其它列席人员提出意见。

　　一、近三年工作回顾

　　本届政府任期近三年来，在中共赣州市委的正确领导下，高举邓小平理论和“三个代表”重要思想伟大旗帜，坚持以科学发展观统领全局，紧紧依靠全市人民，全面实施“对接长珠闽，建设新赣州”发展战略，围绕“一条主线、六个重点”，齐心协力，扎实工作，经济社会发展取得令人瞩目的成就。预计２００６年，全市生产总值达５８２亿元，按可比价计算年均增长１２．５％；三次产业结构由２００３年的２９．４：３２．１：３８．５调整为２３．２：３８．８：３８．０，二产比重提高６．７个百分点；财政总收入突破５６亿元，年均增长２１．３％，占ＧＤＰ的比重由２００３年的８．５７％提高至９．６２％，地方财政收入达到３０．３亿元，年均增长１５．５％；社会消费品零售总额１８４亿元，年均增长１３．４％。

　　新农村建设在全国先行一步，农村面貌明显变化。扎实推进新农村建设，完成２６４个圩镇、３２１８个行政村和３１６７１个村庄的规划编制，改造通村公路５３９３公里，完成７５３４个村庄的改水、改厕工作，整治改造“空心房”７０３．９７万平方米。培训农民１５７．６万人次，“一村一名大、中专生”培养工作顺利进行。组建农村合作经济组织３３５４个，联结农户７０．２万户。建成一批功能齐全的农村社区服务中心。农业产业化水平稳步提升。在稳定发展粮食生产的同时，农业主导产业和区域特色产业不断壮大，脐橙种植面积由７５万亩发展至１２３万亩，肉类总产量年均增长６．１％，农产品商品率达６９％，农产品年销售收入超亿元的龙头企业１６家。预计２００６年全市农业总产值达到２０９亿元，年均增长４．０％。

　　产业培育和结构调整力度加大，工业发展水平明显提升。预计２００６年，全市规模以上工业完成增加值１１０亿元，按可比价计算年均增长２８．４％；销售收入３４５亿元，利税３９亿元，分别增长４５．７％和４２．０％；规模以上工业增加值、销售收入和利税两年翻了一番。钨、稀土和氟盐化工三大优势产业实现销售收入、利税总额分别占规模以上工业的４０％、４６％。有色冶金及新材料、食品、机电制造等六大主导产业完成工业增加值、利税总额占规模以上工业的８２％和８５％。销售收入超亿元或利税过千万元企业可达１００户，６０户重点企业实现销售收入、利税总额分别占规模以上工业企业的４９％和５３％。章源钨制品硬质合金技改、万年青水泥一期、江齿汽车传动器技改等一批工业项目完工。１６个工业园区实现销售收入２８０亿元、利税总额２８亿元，分别是２００３年的３．１倍和２．６４倍。

　　旅游、物流等现代服务业呈现良好发展态势，第三产业增长速度明显加快。预计２００６年第三产业实现增加值２２１亿元，年均增长１４．２％。旅游业快速发展，共接待国内外游客６９８．６９万人次，年均增长１０．４％；旅游总收入４５．６７亿元，年均增长１５．６％。“一个园区、三个中心、三个集散地”的物流体系建设进展顺利。交通运输邮电通信业增加值年均增长７．７％，批发零售住宿餐饮业年均增长１５．７％。金融业有效服务经济建设，各项存款、贷款余额年均分别增长１８．７４％、７．６６％。房地产业稳步发展，增加值年均增长１３．２％。

　　改革开放深入推进，发展活力明显增强。出台“十大体系”１０个主体文件和９３个配套文件。市、县“十大体系”建设扎实有效，实施的１３４个物质载体项目有８６个建成并投入使用。建立了国有资产监管新体制，“大交通”管理格局基本形成，新组建的１６个和职能调整的１０个机构运作良好。强县扩权改革试点初见成效。国家级出口加工区申报工作进展顺利。９２％的事业单位实行全员聘用制。乡镇机构、农村税费和义务教育管理体制等各项农村改革稳步推进，林业产权制度改革基本完成。三年来实际引进内资４７３．０２亿元，实际利用外资１８．３５亿美元，出口１１．７２亿美元。预计２００６年个私企业注册资金１７６．７亿元，上交税金２３．５３亿元，年均分别增长２５．３％和３２．３％，非公有制经济比重比２００３年提高９个百分点。

　　基础设施和城市建设步伐加快，发展环境明显优化。三年累计完成固定资产投资５６７亿元，年均增长１０．１％。赣龙铁路、赣粤高速公路全线贯通，康大高速、赣州机场迁建、居龙滩水利枢纽工程等一批项目正抓紧建设，华能瑞金（赣州）电厂、赣州至瑞金隘岭等四条高速公路、赣韶铁路等项目前期工作进展顺利。三年共改造或新建国、省道７８７公里，改造农村公路７６７３公里。新增发电装机容量１２．２万千瓦，农村电网和７个县（市、区）城网得到改造，赣州５００ＫＶ输电线路和变电站、瑞金至潭东和万安至潭东２２０ＫＶ输变电工程等一批项目竣工。公共保税仓、龙南海关、商检大楼等项目投入运行。完成中心城区３７．１６公里防洪堤建设，已安排２１９座病险水库的除险加固，新增有效灌溉面积１１．６８万亩，改造中低产田４５．１２万亩。三年来，全市共完成市政公用基础设施建设投资３９．８３亿元，中心城区建成区面积达４３平方公里，全市城镇化率达到３３．１％，比２００３年提高６．２个百分点。中心城区完成１６项规划编制，改造２９条主次干道和５７条小街小巷，完成古城墙、东河大桥、红旗大道绿化亮化及整饰工程，建设“１２３”人防工程、青少年活动中心、市传染病医院等一批市政公共设施项目，中心城区日供水能力由１７万吨提高到２７万吨。

　　关注民生工作深入扎实，人民生活明显改善。预计２００６年，城市居民人均可支配收入９１４０元，年均增长１０．８％；农民人均纯收入３０００元，年均增长１０．２％。城乡居民储蓄存款４５５亿元，年均增长１９．２１％。年均新增城镇就业人员７．４０万人，困难群体再就业人数年均增长１５．３％，城镇登记失业率控制在３．４％以内。社会保险覆盖面扩大到１１４．５万人，比２００３年新增参保人员２５．８万人，企业离退休人员养老金确保按时足额发放。初步建立以“十大救助制度”为主要内容的城乡社会救助体系和农村低保制度，１０．２万人享受城市低保，２０．１４万人享受农村低保。新建灾民新村１３９个，灾后倒房重建率达到１００％，五保集中供养率达到６５％。新型农村合作医疗试点覆盖人口由三年前的１５万提高到２５８．２万。１４个县（市、区）启动廉租房建设，中心城区完成４．８５万平方米，开工在建５．１４万平方米。解决了２９．５万农村人口的饮水不安全问题。减少贫困人口３．０２万人，完成深山区、库区、地质灾害区群众移民搬迁４．３８万人。

　　科技教育快速发展，各项社会事业明显进步。三年来获省以上科技奖１２项，申请专利５５０项，高新技术企业和生产高新技术产品企业达４８家，初步建立脐橙、稀土、钨等优势产业的技术标准体系。“两基”目标全面实现，全市小学适龄儿童入学率稳定在９８％左右，初中升高中学生比例达到５５％，比２００３年提高１０个百分点。职业教育和民办教育快速发展。人才资源开发步伐加快，全市人才总量达到５８．６１万人。成功举办第１９届世界客属恳亲大会、客家文化节、中国（江西）红色旅游博览会、脐橙节等活动，文艺创作和演出在省以上获奖项目增多。全民健身活动广泛开展，竞技体育水平大幅度提高。建立健全市、县两级突发公共卫生事件应急指挥机制和救治体系，县级疾控中心和传染病区项目建成并全部投入运行。完成国家和省定７４７个盲点村与９４个返盲村的“村村通”建设任务，全市广播、电视综合覆盖率达９５％和９６％。广泛开展创建文明城市、文明社区、文明单位活动，推广实施“留守孩关爱工程”。人口出生率比２００３年下降了０．８２个千分点，人口自然增长率下降０．４个千分点。两次顺利通过国务院土地市场秩序治理整顿检查验收。矿产资源得到有效保护，三年完成水土流失综合防治面积１７．２４万公顷，城区大气质量、主要河流水质等环保指标保持较好水平，森林覆盖率达到７４．２％。社会治安综合治理连续三届被评为全国先进，并荣获全国首届“长安杯”。道路交通、食品药品、矿山和危险化学品安全等专项整治深入开展。信访工作机制进一步完善。“四五”普法全面完成。民兵预备役建设、国防动员和人民防空工作取得显著成绩。“双拥”共建活动和优抚工作取得较好成效，获省“双拥”模范城市称号。监察、审计、物价、统计、水文、气象、档案、防震减灾、民族宗教、对台和外事侨务、新闻出版、地方志、老龄、妇女儿童、残联等各项工作都取得了新的成绩。

　　政府自身建设不断加强，行政能力明显提高。坚持并完善向人大报告工作、向政协通报情况制度，自觉接受人大、政协的监督，三年来共办理人大代表建议５２６件、政协委员提案８８０件。贯彻实施《行政许可法》和国务院《全面推进依法行政实施纲要》，行政执法责任制全面推行，行政复议工作得到加强。颁布了《政府信息公开暂行规定》，建立了政府新闻发布工作制度，电子政务建设取得较大进展。初步建立突发公共事件应急管理体系。市、县两级行政服务中心功能进一步完善。认真开展保持共产党员先进性教育活动，组织机关干部参与和指导新农村建设。深入开展优化发展环境机关效能监察工作，纠风和专项治理取得实效。惩防体系建设初见成效，党风廉政建设和反腐败工作得到加强。

　　各位代表，过去的三年，是我市自上世纪九十年代中期以来经济增长最快，发展质量最好，居民收入增加最多的时期。历程难以忘怀，成绩来之不易。在此，我谨代表市人民政府，向付出辛勤劳动的全市广大工人、农民、知识分子和干部职工，向给予政府工作大力支持的市人大代表、政协委员，向驻市人民解放军、武警官兵、公安民警和离退休老同志，向各民主党派、工商联、人民团体、社会各界人士以及所有关心支持赣州现代化建设的海内外朋友们，表示崇高的敬意和衷心的感谢！

　　在看到成绩的同时，我们也清醒地认识到发展中存在的困难和问题，主要表现为：经济总量小、人均水平低的欠发达市情没有根本改变；产业规模小、层次低、竞争力不强，仍处于工业化初级阶段，加快发展与科学发展的压力较大；中心城市带动力不足，对接平台不完善，市场环境不够优化等问题依然存在；农民持续增收、城乡协调发展、人与自然和谐发展的任务依然艰巨；政府自身建设有待加强等。这些困难和问题，有待于在今后的工作中，采取切实有效措施逐步加以解决。

　　二、今后五年的发展目标和主要任务

　　综观今后五年，我们面临总体趋好的发展环境。经济全球化和区域经济一体化趋势加速，科技进步日新月异，生产要素重组和产业分工转移步伐加快，我国宏观调控更加完善，国民经济将进入新一轮持续稳定增长期。我市经过多年特别是近三年来的努力，进一步理清了发展思路和战略重点，积蓄了发展能量和后劲，后发优势日益显现。与此同时，经济运行中的不确定、不稳定因素增多，长珠闽等地区发展态势强劲，区域竞争更趋激烈，资源约束和环境压力越来越大。我们一定要抢抓发展机遇，积极应对挑战，趋利避害，奋发图强，坚持科学发展不动摇，努力实现国民经济又好又快发展。按照市第三次党代会的总体部署，未来五年，我们要以邓小平理论和“三个代表”重要思想为指导，全面落实科学发展观，纵深推进“对接长珠闽，建设新赣州”发展战略，创新体制机制，扩大对外开放，转变增长方式，统筹城乡发展，促进社会和谐，全面加速农业农村现代化、新型工业化、新型城镇化和经济市场化，不失时机地推进信息化和经济国际化，实现经济社会的追赶和跨越，在江西的崛起中率先发展，基本确立赣粤闽湘四省通衢的区域性现代化中心城市地位，为全面建设小康社会奠定更为坚实的基础。

　　未来五年的主要目标是：全市生产总值到２０１１年翻一番，力争达到１２００亿元；财政总收入到２０１１年翻一番，力争达到１２０亿元；固定资产投资年均增长２０％；社会消费品零售总额年均增长１２％；实际利用外资和引进市外资金年均分别增长１０％，外贸出口年均增长２０％；城市居民人均可支配收入年均增长９％，农民人均纯收入年均增长８％；居民消费价格总水平年均控制在１０３％以内；单位生产总值能耗明显降低；人口年均自然增长率控制在８．２‰以内；城镇登记失业率控制在４％以下；就业、社保、科技、教育、文化、卫生、体育、环保等社会事业取得新的发展。

　　未来五年，我们要纵深推进“对接长珠闽，建设新赣州”发展战略，实现赣州经济社会科学和谐的追赶型、跨越式发展，坚定不移地抓好经济社会发展的五个重点，着力形成五个新格局。

　　坚定不移地抓好“十大体系”建设这个重点，深化改革开放，逐步形成与长珠闽特别是珠三角对接互动的更高阶段和更高效益的开放新格局。进一步完善政策法规、金融服务、信息网络、人力资源、物流服务、贸易服务、产权交易、口岸服务、技术标准、社会保障等“十大体系”的政策框架和物质载体，推动制度创新成果迅速转化为加快发展的强大动力，加大重要领域和关键环节的改革攻坚力度，在全省率先完善社会主义市场经济体制，基本形成与长珠闽特别是珠三角对接互动的新机制。将赣州建设成为对接粤港澳的“桥头堡”，成为吸纳投资、集聚财富、汇集人才的全方位开放城市，提高开放型经济对经济增长的贡献率。

　　坚定不移地抓好社会主义新农村建设这个重点，壮大农村和城镇经济，逐步形成特色更加鲜明、实力更加雄厚的县域经济发展新格局。按照生产发展、生活富裕、乡风文明、村容整洁、管理民主的总体要求，以促进农村生产力发展和农民增收为重点，不断探索有赣州特色的工作机制和路径，发展新产业，建设新村镇，培育新农民，组建新经济组织，塑造新风貌，创建好班子，扎实稳步建设社会主义新农村。着力壮大城镇经济，努力把县城和重点集镇建设成具有生产、贸易、交通、居住、消费等多种功能的县域经济增长中心。立足各地的比较优势，积极发展特色经济，形成各具特色的县域主导产业，培育壮大一批经济强县，打造一批特色鲜明的经济板块。

　　坚定不移地抓好培育优势产业集群这个重点，提升产业发展水平，逐步形成链条更长、附加值更高、竞争力更强的新型工业化格局。充分发挥比较优势和后发优势，把工业化与信息化、生态化结合起来，加快转变增长方式，扩大有效总量，提升发展质量和效益，把钨、稀土、氟盐化工、脐橙等优势产业建成产值超百亿元的产业集群。加快把赣州建设成为国际知名的钨、稀土加工基地，全国重要的钴、锡等有色金属产业区和汽车变速器生产基地，全省重要的氟盐化工基地，以及全国最大、世界著名的脐橙主产区。到２０１１年，规模以上工业销售收入迈上１０００亿元台阶，三次产业结构比达到１５∶４８∶３７左右。

　　坚定不移地抓好基础设施和城镇化建设这个重点，增强城市集聚辐射能力，逐步形成赣粤闽湘四省通衢的区域性现代化中心城市新格局。加快以高速公路为重点的大交通建设，完善能源、信息等基础设施。不断增强中心城区的产业聚集能力、人口吸纳能力、要素集约能力、创新发展能力和辐射带动能力，到２０１１年，中心城区面积达到７０平方公里，人口７０万以上，基本确立赣粤闽湘四省通衢的区域性现代化中心城市地位。把瑞金、龙南两个次中心城市发展成为中等城市，形成瑞金——于都——中心城区——南康——信丰——龙南城市带，全市城镇化率达到４２％以上。

　　坚定不移地抓好和谐平安赣州建设这个重点，以人为本、关注民生，逐步形成人民生活更加殷实、社会更加文明安定的和谐发展新格局。认真解决人民群众最关心、最直接、最现实的利益问题，全面发展各项社会事业，扩大社会保险覆盖面，促进城乡充分就业和社会公平。不断提高人民群众的思想道德素质、科学文化素质和健康素质，进一步形成和谐人际关系和良好道德风尚。树立生态立市、绿色发展的理念，打造绿色生态赣州，建设资源节约型和环境友好型社会。提升人民群众的生活质量和幸福指数，增强公众安全感、幸福感和社会和谐度。

　　三、２００７年政府工作的主要安排

　　２００７年是新一届政府施政的开局之年，做好明年的政府工作意义重大。全市经济和社会发展的主要预期目标是：生产总值增长１２．５％，财政总收入增长１３％，地方一般预算收入增长１１％，实际利用外资增长１０％，引进内资增长１０％，固定资产投资增长２０％，城镇居民人均可支配收入增长９．４％，农民人均纯收入增长８％，城镇登记失业率控制在４％以内，人口自然增长率控制在８．３‰以内。为实现上述目标，重点抓好八个方面的工作：

　　（一）集中力量主攻项目，在推进重大项目建设上有新作为

　　拓宽项目策划和开发思路，大力开发和储备一批符合国家产业政策和扶持方向的项目，建立市级项目库。做好项目的信息收集、分析、论证、审批、核准等工作。实行重大项目建设领导联系制度、定期调度制度、现场办公制度，加大对项目建设的推进力度。强化对项目建设质量、工期、资金和招投标等方面的监督管理，顺应重大项目投资多元化趋势，建立健全重大项目建设的准入机制、奖惩机制和优惠政策体系。全年计划安排市重点工程３０个，完成投资７４亿元。交通方面加快赣州机场迁建、赣韶铁路、赣州至大余高速公路、赣江大桥等项目建设，完成国省道和市养公路２０３公里，农村新建水泥（油）路２５００公里；能源方面启动华能瑞金（赣州）电厂建设，加快居龙滩电站等项目建设；城建方面抓好污水处理厂和飞龙岛大桥等项目建设；社会事业方面抓好赣州卫校整体搬迁、博物馆、游泳馆等项目建设；工业方面抓好华茂钨材公司钨深加工、赣南果业酒业分公司异地技改等项目建设，工业重点项目投资力争完成２０亿元以上。同时，抓好一批招商引资、城市建设和农业产业化等方面的重大项目建设。

　　（二）不断深化改革开放，在增强经济发展内生动力上有新作为

　　深入推进“十大体系”建设。坚持抓落实、抓完善、抓转化，创优发展环境，推进“十大体系”政策框架和物质载体建设。贯彻执行已出台的各项政策文件，对新组建和调整职能的２６个机构加强跟踪问效，开展“十大体系”进工业园区等系列活动。引导县（市、区）全面推进“十大体系”建设，建立和落实政策效应评估及督促考核机制。用好国家中部崛起和西部大开发政策，继续借鉴吸收长珠闽尤其是珠三角等地的新鲜经验，进一步修订完善或出台新的配套政策，不断充实和创新“十大体系”建设内容。

　　大力发展开放型经济和民营经济。把全面开放、全民创业作为促进我市经济社会追赶型、跨越式发展的两个战略支点，坚持对内对外开放并举，促进外源型发展与内源型发展紧密结合。强化招商引资的领导和组织力量，广泛开展形式多样的招商活动，举办和组织好各项重大招商活动。立足资源和产业优势，吸引一批国内外战略投资者特别是中央直属大企业、大集团到赣州投资，引进一批完善产业配套的项目，主攻一批５０００万元以上的工业项目，承接一批沿海梯度转移项目。加强对重点地区、重点企业的招商，增强招商的针对性和选择性，变招商引资为招商选资，变政策招商为环境招商，提高招商质量和效益。抓好招商队伍的培训，完善招商引资工作的调度、督查和考核。及时帮助招商引资企业解决招工难、用地难、融资难等问题。鼓励和支持企业扩大外贸出口，力争全年完成出口６亿美元，增长２０％。深入推动全民创业，浓厚创业氛围，激活各类创业主体，完善和落实鼓励全民创业、支持民营经济发展的政策措施，优化创业环境，为民营企业搞好信息网络、创业培训、信贷融资、知识产权等方面的服务。建立一批创业基地，孵化一批民营小工业企业，培育一批民营企业上规模、上水平。

　　（三）整体推进“五新一好”，在建设社会主义新农村上有新作为

　　加快农村经济发展。力争经济作物产值占种植业总产值的６０％以上，养殖业产值占农业总产值的６０％以上。稳定发展粮食生产，粮食总产达到５２亿斤以上。实施“一乡一业”、“一村一品”发展战略，重点抓好５０个乡镇、５００个村的产业示范工作。发展壮大主导产业和区域特色产业，提升脐橙产业发展规模和水平，新增脐橙１０万亩，力争出栏生猪５００万头，蔬菜面积达到１６５万亩，花卉面积达到５万亩。大力发展农产品加工、流通业，扶持农业龙头企业，争取５０％以上农民自愿加入各类农民专业合作社。推进科技兴农，培训１万名农村科技带头人，选择推广１０项重大技术，加快农业机械化进程。加强农产品商标和地理标志证明商标申报注册，形成产业品牌优势。

　　搞好新型村落社区建设。市本级财政明年安排新农村建设的资金在今年的基础上增加１５００万元，以后年度按不低于经常性财政收入增长幅度安排。做好乡镇总体规划，探索圩镇规划建设管理新模式，建设一批有客家文化特色的新村落。全面开展“三清三改”和村庄整治，解决圩镇脏乱差问题。扩大新农村建设的覆盖面，新农村建设点达到３５００个。积极推进布局优化、道路硬化、村庄绿化、卫生洁化、河道净化、管理长效化等“六化”建设，加快农田水利、道路、通信等农村基础设施建设，强化农村防汛减灾、森林防火、动物防疫等队伍建设，保障农业安全。深化农村综合改革，大力发展农村社会事业。建立新型农村自治机制，提升农村人居环境和农村社会文明。

　　（四）着力培育产业集群，在壮大工业经济实力上有新作为

　　坚持扶优扶强，加快优势产业集群、主导产业和重点企业发展。实施资源导向型和市场导向型并重的工业发展战略，努力实现规模以上工业增加值、销售收入、利税总额分别增长２２％、２５％、２０％，工业占ＧＤＰ比重力争提高２个百分点，工业万元产值综合能耗比上年下降的奋斗目标。做好产业集群和产业基地规划，加大资源整合力度，支持产业链龙头企业发展，推动钨、稀土、氟盐化工三大优势产业集群升级发展，总体增长２６％，其中钨和稀土产业销售收入分别达到１１５亿元和５０亿元。综合运用财政、信贷、投资等方面政策，扶持发展食品、现代轻纺、机电制造、新型建材、制药等主导产业，打造多个产业增长极。大力培育产业龙头，全市重点调度１００户重点企业，销售收入超亿元或利税超千万元企业净增２０户以上，规模以上企业净增５０户以上。

　　加快经济技术开发区和工业园区建设，提高产业承载和集聚能力。集中项目、集中客商、集中力量，支持经济技术开发区加快发展。加大力度主攻工业，重点打造有色产业基地，搞好出口加工区申报和建设，完善基础设施和物流配送、劳动用工、信息咨询等产业配套。加大力度主攻优势产业，以发展高附加值的深度加工和应用产业为主线，在引进外商外资、国企国资、民企民资上有大的突破，重点引进投资１亿元以上项目和有色金属深加工项目，形成产业发展的“洼地”。加大力度主攻核心企业，整合优势资源向优势企业集中，争取引进更多的国内外大公司大集团，搞好已落户企业和项目的服务。充分发挥全市１６个工业园区主攻工业的平台作用，使园区成为工业发展的主引擎。

　　（五）突出发展旅游、物流业，在繁荣第三产业上有新作为

　　进一步完善旅游基础设施和配套设施，加快１３个核心旅游区建设，重点调度瑞金、上犹陡水湖、安远三百山、主城区等旅游景区建设，创建４个４Ａ级旅游景区、１００个新农村旅游示范点，打造“千里自驾走廊”，做大做强红色故都、江南宋城、客家摇篮、东江源头四大旅游品牌，把赣州建设成为粤港澳地区的后花园。推进“一个园区、三个中心、十大工程”的物流体系建设，重点抓好综合物流园区、物流配送中心和物流信息平台建设，积极引进和发展综合型、配送型、加工主导型等现代物流企业。着力优化金融生态环境，吸引更多金融机构到我市落户。做大做强地方金融机构，支持市商业银行增资扩股和延伸机构，加快农村信用社改革和发展。改造提升商业、贸易、交通、运输、餐饮、娱乐等传统服务业，发展特许经营、连锁经营、加盟经营等新型业态。完善房地产市场体系，健康发展房地产业。综合开发利用信息资源，大力发展信息服务业。不断壮大文化产业，加快发展法律、会计、咨询评估、广告策划、健康服务等城市服务业，扶持农村服务业发展。全市服务业增加值达到２６０亿元，增长１４．５％。

　　（六）提升城镇化发展水平，在中心城市建设上有新作为

　　树立贴近时代、贴近市情、贴近市民的“三贴近”建设理念，确立打造核心、张扬个性、塑造特色的建设思路，以国家卫生城市、园林城市、“双拥”模范城市“三城”同创为抓手，做大做强做美中心城区。拓展城市框架，新增城市道路面积５０万平方米，新增公共绿地５０万平方米。进一步拉开经济技术开发区建设框架，加大新城区统筹发展力度，加快完善创新区基础设施，抓紧规划和实施整体开发黄金机场及周边地区建设项目。完善城市功能，建设文峰路北段、兴国中路、黄金三路、章江浮桥等一批市政项目。优化河套老城区功能，力争两年内完成８０１厂、气压机厂等老企业“退城进园”，改造赣江路危旧房，整治南市街、灶儿巷、姚衙前。加强新城区功能配套，抓好新城区给排水、燃气、电力通信、防空设施等市政设施和学校、医院、环卫设施、商业网点、停车场等生活配套设施建设。提升城市品位，结合历史文化名城保护和历史街区修缮，重点抓好迎宾大道、３２３国道、宋城路等城市进入口交通和景观改造，优化郁孤台、八境台周边景观。创新城市管理机制，推行建管分开、管养分离和委托承包治理。以扩绿增绿为重点，新建栎木坑、杨梅渡公园，全面启动空间立体绿化工程。加大创建园林城市工作力度，争取中心城区成功申报国家园林城市。强化城市经营理念，重新认识城市，重塑城市价值。完善土地收储和经营机制，吸引外资、民资进入城市供水、燃气、公交、污水处理、道路与桥梁等市政公用行业，加快形成多元化的城市建设投融资机制。推进县城及中心镇建设，形成一批特色小城镇，力争全市城镇化率提高２个百分点以上。

　　（七）推进科技教育创新，在发展各项社会事业上有新作为

　　加快科技、教育事业发展。以高新技术产业开发区为核心，加强区域创新体系建设，支持和鼓励企业建立技术研发机构、研究中心或博士后流动站。建立技术创新孵化基地，提高省级特色产业基地科研能力，积极申报国家级新材料产业基地。全面实施《义务教育法》，巩固提高九年义务教育水平和质量。进一步完善“以县为主”的义务教育管理体制，发展学前教育，实施素质教育，完善“优质高中建设工程”，提升高等教育质量。大力发展职业教育和成人教育，推进现代远程教育，重视社区教育，规范和促进民办教育发展。继续推进“公民职业化”和“农民知识化”工程。进一步完善人力资源体系，突出创新型、技能型、应用型人才的培养和引进，做好非公有制经济和农村人才的开发工作，选拔录用３５０名优秀大学生到基层锻炼。

　　全面发展文化、卫生、体育等社会事业。抓好公益性文化设施建设，加强圩镇文化示范点规划建设，建立非物质文化遗产保护制度。大力发展和建设和谐文化、创业文化，广泛开展各种形式的群众性文化活动，举办第二届社区文化艺术节和全市农民艺术节，培育和扶持群众文艺社团。加强公共卫生体系建设，加大重大疾病防治力度，基本建成较为完善的城市社区卫生服务体系，提高城镇医疗卫生技术和服务水平。加快已通电２０户以上自然村广播电视工程建设，扩大广播电视在农村的有效覆盖。办好第二届市运会，继续提升竞技体育水平。实施妇女、儿童发展规划，关心和支持老龄与残疾人事业。认真开展好全国第二次农业普查。继续做好国防动员、民兵预备役和双拥工作。进一步抓好对台和外事侨务、统计、物价、审计、防震减灾、人防、档案、地方志、红十字会等工作，推动各项社会事业全面发展。

　　加强精神文明和民主法制建设。树立以“社会主义荣辱观”为主要内容的社会主义荣辱观，在全社会形成知荣辱、讲正气、促和谐的风尚。开展文明城市、文明村镇、文明行业创建活动。完善爱国主义教育基地、城乡精神文明活动场馆建设，做好各级文明单位与文明村镇结对帮扶工作。自觉接受人大依法监督、政协民主监督和社会监督，主动加强与各民主党派、工商联、人民团体及无党派人士的联系，认真做好人大代表建议和政协提案办理工作。广泛开展法制宣传教育，推进依法治市。

　　（八）重视解决民生问题，在构建和谐社会上有新作为

　　关注民生、为民办事。把发展经济与改善人民生活统一起来，做到发展为了人民，发展依靠人民，发展成果由人民共享。每年新增财力继续重点向困难群体倾斜，向农村倾斜，向基层倾斜，向社会事业倾斜，着力发展普惠型事业，切实为人民群众办实事、解难事、做好事。１、新增城镇就业８．５万人，引导转移农村劳动力达到１６０万人，帮助３．７万下岗失业人员实现再就业，发放小额贷款１．６亿元，加快乡镇劳动保障事务所建设。２、扩大社会保险覆盖面，力争达到１２５万人；完善城乡低保，逐步提高五保集中供养率。３、全面完成中心城区小街小巷整治，加快经济适用房和廉租房建设，中心城区经济适用房建设规模达到２３万平方米，廉租房施工面积达到７万平方米。４、建立健全覆盖城乡的基本医疗保障体系，力争社区卫生服务网络覆盖９５％以上的城镇居民；新增７个新型农村合作医疗试点县（市），新增覆盖农村人口２７５．８万人。５、加大学校危房改造力度，免除城乡义务教育阶段学杂费，对贫困家庭学生免费提供教科书并补助寄宿生生活费。６、加快乡镇文化站、影剧院等文化阵地建设，培育一批特色文化村、文化室和文化中心户；加强农村体育设施建设。７、新建山塘２２５座以上，新建或维修农村机电泵站（机井）及提水设备６９０座（处），基本完成规划内２５１座病险水库除险加固，完成新农村建设改水人口５０万人。８、抓好扶贫贷款贴息到户，力争１．６万户贫困户受益。９、做好１．５万人的易地扶贫搬迁工作，力争全年减少贫困人口１万人。１０、着力解决好民族村通电、通水泥路、通电话、通电视和用上卫生饮用水问题，改善民族村医疗卫生条件。

　　做好人口、资源和环境保护工作。严格落实现行生育政策，稳定低生育水平，遏止出生人口性别比持续升高势头，提高出生人口素质，坚决执行计划生育“一票否决”制。深入开展矿产资源开发秩序整顿和规范工作，建立依法保护、有序开发、合理利用、和谐发展的矿业经济发展模式。依法强化土地管理，严格保护耕地，节约集约用地，坚决查处土地违法违规行为。强化水资源和林业资源管理，加快水土流失治理。严格控制污染物排放总量，狠抓工业污染源治理。

　　加强社会建设与管理。整合社会管理资源，提升社会管理水平。加强和改进新形势下的群众工作及信访工作，形成科学有效的利益协调机制、诉求表达机制、矛盾调处机制、权益保障机制，畅通信访渠道，切实减少非正常上访。积极推进城市社区和农村村落社区建设，完善基层服务和管理网络。切实贯彻民族宗教政策，促进民族关系、宗教关系的和谐。进一步落实安全生产责任制，严肃查处安全生产领域的违法违纪行为，继续确保“两杜绝、一减少”目标的实现。完善各项应急预案，提高保障公共安全、处置突发事件和抗击灾害事故的能力。深入开展和谐平安赣州创建活动，全面推进社会治安综合治理，保持社会安定有序。

　　四、加强政府自身建设

　　在推进赣州经济社会科学和谐的追赶型、跨越式发展征途上，新形势、新任务对政府工作提出了新的更高的要求，我们将紧紧围绕“三个提高、一个创新”，建设创新、高效、透明、诚信政府，不断提高行政能力和水平。

　　提高政府执行力。大力倡导政府执行文化，强化“执行没有借口”理念，接受任务不讲条件，执行任务不找借口，完成任务追求圆满。全面实施《公务员法》，加强公务员队伍建设、管理和培训，弘扬公务员精神，建设学习型机关和学习型干部队伍，切实提高政府机关及公务员的执行能力。建立纵向到底、横向到边的工作责任体系，细化分解目标任务，按各自工作分工明确责任主体，量化责任内容，确保各项工作落到实处。严格执行行政问责制，对已明确的任务、确定的事项，既责任到人，又问责到位，做到有权必有责、有责必问责、有错必追究，严肃查处“不作为”、“乱作为”、失职渎职行为，杜绝推诿扯皮，着力解决无人执行、无力执行、无心执行的问题，确保政令畅通。奖勤罚懒，用能去庸，支持和重用敢抓敢管、认真负责、干事创业的干部，着力提高执行效率和效果，建设高效政府。

　　提高政府公信力。大力弘扬实干精神，以大干、善干、干好、干成来成就事业，赢得民心，树立形象。完善重大问题集体决策、专家咨询、社会公示听证、决策效果评估制度，提高政府决策的民主化、科学化水平。严格推行行政执法责任制，确保政府各部门和公务员按照法定权限和程序行使职权、履行职责。整顿和规范市场秩序，严厉打击制假售假、合同诈骗、恶意拖欠等违法行为，维护公平正义。增强公务员的诚信观念，加强政府信用建设，使各级政府恪尽职守，有诺必践，讲究信用。加大失信惩戒力度，在全社会营造诚信为本、操守为重的良好风尚，打造诚信政府，建设诚信赣州。

　　提高群众满意率。把利民、便民、惠民、富民作为政府工作的基本准则，花更多的精力、用更多的财力来解决就业、就学、就医、社保等民生问题。在经济建设中更加注重环境优化，在改革改制中更加注重平稳有序，在征地拆迁中更加注重合法维权，在工程建设中更加注重方便群众。认真执行首办负责制、限时办结制、服务承诺制、全程代理制、失职追究制，做到用制度管权、按制度办事、靠制度管人。强化行政监察和审计监督，推进惩治和预防腐败体系建设，严格落实廉政建设责任制，坚决纠正损害群众利益的不正之风。加强行政服务中心建设，行政审批职能集中到窗口、授权到窗口、办结在窗口，把行政服务中心建设成行政便民“超市”。推进政务公开，推行阳光政务，完善新闻发布工作制度，提高行政权力运行透明度，保障公民的知情权、参与权和监督权，建设透明政府。

　　创新政府管理。进一步转变政府职能，实行政企分开、政资分开、政事分开以及政府与市场中介组织分开。减少政府对经济事务的微观管理和对经济社会活动的直接干预，发挥行业协会、商会和社会中介组织的作用，有效运用规划、产业政策、技术和环境标准等手段管理经济。优化部门工作流程，推行电子政务，降低行政成本。进一步理顺政府部门的职能分工，合理划分事权、财权，实现职能、机构、编制的科学化和法定化。积极探索建立政府部门绩效评价指标体系，对政府部门履行法定职责、完成工作目标任务、使用财政资金、依法行政以及工作效率等方面的情况进行综合考评，努力建设创新政府。

　　各位代表，新的征程催人奋进，新的使命光荣神圣。让我们更加紧密地团结在以胡锦涛同志为总书记的党中央周围，高举邓小平理论和“三个代表”重要思想伟大旗帜，全面落实科学发展观，在中共赣州市委领导下，同心同德，开拓创新，励精图治，艰苦创业，为建设赣州更加美好的明天而努力奋斗！
